# Early diagnosis of radiation-induced renal injury: from lagging indicators to multimodal, pre-fibrotic detection

**DOI:** 10.3389/fcell.2026.1869203

**Published:** 2026-09-03

**Authors:** Yuanyuan Zhang, Eric P. Cohen, Sean V. Murphy, Tarek Zaho, Hongbing Liu, Heather Himburg, Michael Patrick Feloney, George William Schaaf, Anthony Atala, J. Mark Cline

**Affiliations:** 1 Wake Forest Institute for Regenerative Medicine, Wake Forest University School of Medicine, Winston-Salem, NC, United States; 2 Department of Urology, Wake Forest University School of Medicine, Winston-Salem, NC, United States; 3 Department of Medicine (Nephrology), New York University School of Medicine, New York, NY, United States; 4 Department of Pediatric Nephrology, School of Medicine Tulane University, New Orleans, LA, United States; 5 Department of Radiation Oncology, Medical College of Wisconsin, Milwaukee, WI, United States; 6 Department of Urology, Creighton University School of Medicine, Omaha, NE, United States; 7 Department of Pathology (Comparative Medicine), Wake Forest University School of Medicine, Winston-Salem, NC, United States

**Keywords:** cystatin C, kidney injury molecule-1, MAG3 renography, multiparametric MRI, NGAL, radiation nephropathy, radiation-induced renal injury, renal elastography

## Abstract

**Background:**

Radiation-induced renal injury (RRI) is characterized by a prolonged latent phase from radiation exposure to clinical dysfunction. Conventional markers such as serum creatinine and blood urea nitrogen (BUN) may only increase after substantial nephron loss, thereby missing a critical therapeutic window during which renal injury may remain amenable to mitigation.

**Objective:**

This review synthesizes emerging strategies for the early, pre-fibrotic detection of RRI, emphasizing molecular damage biomarkers, genomic and epigenetic indicators, and advanced functional imaging techniques that may identify subclinical abnormalities before irreversible fibrosis develops.

**Content:**

Tubular damage biomarkers such as kidney injury molecule-1 (KIM-1) and neutrophil gelatinase-associated lipocalin (NGAL) provide sensitive signals that precede functional decline. Filtration markers such as cystatin C may improve early risk stratification compared with creatinine alone. Genomic indicators, including γ-H2AX foci and circulating or urinary microRNAs such as miR-21 and miR-29 capture DNA damage and fibrotic signaling transitions. Advanced imaging modalities—particularly non-contrast multiparametric MRI (diffusion-weighted imaging/intravoxel incoherent motion, arterial spin labeling, blood oxygen level–dependent MRI, and T1 mapping)—demonstrate strong correlations with biopsy-proven fibrosis and microvascular dysfunction. Tc-99m MAG3 renography further reveals split renal function and asymmetrical injury masked by preserved contralateral reserve.

**Conclusion:**

Evidence supporting these candidate approaches in human radiation-induced renal injury remains limited. Tubular biomarkers, cystatin C, genomic indicators, urinary extracellular-vesicle signatures, and multiparametric MRI have demonstrated potential in other renal disorders or preclinical radiation models, but their RRI-specific kinetics, thresholds, sensitivity, and specificity remain insufficiently established. We therefore propose a multimodal research framework integrating radiation exposure and renal dosimetry, serial renal function assessment, candidate biofluid biomarkers, and functional imaging. Prospective longitudinal validation is required before this framework can be used for routine clinical diagnosis or treatment selection.

## Introduction

Radiation-induced renal injury (RRI) represents an insidious yet substantial sequela of sufficient ionizing radiation exposure (Dawson et al., 2010). Its causes include external-beam radiotherapy (EBRT) for abdominal or pelvic malignancies, myeloablative conditioning for hematopoietic cell transplantation (HCT) that has used total body irradiation (TBI), and emerging targeted radionuclide therapies (TRT) (Kal and van Kempen-Harteveld, 2006; Miralbell et al., 1996; Steinhelfer et al., 2024). Even exposures in outer space may result in renal radiation injury (Siew et al., 2024). Although the exact incidence of radiation-induced renal injury (RRI) varies depending on exposure type and dose, clinically significant radiation nephropathy has been reported in up to 5%–15% of patients receiving abdominal radiotherapy involving the kidneys, with higher rates observed in hematopoietic cell transplantation settings following total body irradiation (Dawson et al., 2010). Despite its potential for progression to end-stage renal disease (ESRD), RRI remains a frequently under recognized pathology. This can happen because of the kidney’s protracted latent period following radiation exposure, where subclinical microvascular rarefaction and interstitial fibrosis evolve silently over months or years before manifesting as overt clinical dysfunction (Klaus et al., 2021).

The pathophysiology of RRI is defined by a cascade of damage that converges on a common terminal pathway, regardless of the radiation source or dose distribution. The initial radiolytic insult triggers an endothelial dysfunction then microvascular injury leading to capillary loss and chronic tissue hypoxia, tubular epithelial stress by activation of pro-inflammatory and pro-fibrotic signaling pathways (e.g., TGF- β, Smad signaling), and chronic hypoxia with fibrosis, a self-sustaining cycle in which capillary rarefaction exacerbates hypoxia, further driving myofibroblast activation and collagen deposition. The kidney’s substantial functional reserve acts as a clinical mask; traditional markers, such as serum creatinine and the derived estimated glomerular filtration rate (eGFR), can remain within the normal range until a critical threshold of nephron loss is surpassed. By the time these lagging indices reflect impairment, there may be irreversible fibrosis (Klaus et al., 2021; Ahmad et al., 2023; Verginadis et al., 2025).

The temporal evolution of RRI follows a characteristic sequence. Initial molecular injury, including DNA damage and endothelial dysfunction, occurs within hours to days following radiation exposure. This is followed by a clinically silent latent phase lasting weeks to months, during which microvascular rarefaction, tubular stress, and hypoxia progressively develop. Functional impairment, reflected by reductions in glomerular filtration rate, typically emerges months later, while overt fibrosis and irreversible structural damage may take months to years to fully manifest. Importantly, these phases overlap, creating a critical therapeutic window during the latent phase where early detection strategies may enable intervention before irreversible fibrosis occurs (Klaus et al., 2021; Ahmad et al., 2023).

In this review, the pre-fibrotic window is defined operationally rather than by a universal fixed time interval. It refers to the period following renal radiation exposure during which molecular, endothelial, tubular, perfusion, or oxygenation abnormalities may be detectable, while sustained loss of glomerular filtration and established fibrotic remodeling remain absent. The duration of this period is expected to vary according to radiation dose, fractionation, dose distribution, exposure type, baseline renal reserve, concomitant nephrotoxic treatment, and comorbid disease. Consequently, the proposed window should be regarded as a testable biological state rather than an established clinical time threshold.

In contrast to the acute clinical presentations of radiation-induced lung (pneumonitis) (Rosiello and Merrill, 1990), bowel (enteritis) (Harb et al., 2014), or bladder (cystitis) injury (Yang et al., 2024), which generally receive immediate clinical attention, RRI is characterized by its “slow and silent' progression (Klaus et al., 2021). This temporal dissociation between exposure and functional decline contributes to more difficult investigation compared to other organ injuries. However, the prevention of RRI-induced chronic kidney disease (CKD) and associated cardiovascular morbidity necessitates a shift in approach (Zaho et al., 2025; Shatta et al., 2025).

Contemporary strategies are moving toward early detection, seeking to identify the molecular and structural signatures of RRI during the latent window before initiation of fibrotic processes. This is justified due to the success of mitigation of experimental radiation injury, in which the use of angiotensin-converting enzyme inhibitors (ACEi) during the interval of 3–10 weeks after irradiation exerts a long term benefit to preserve renal function (Cohen et al., 1997). This interval corresponds to the latent window referred to above and implies that there are active mechanisms of later injury that have started during the latent window. In this multimodal approach we stress the identification of early tubular stress and podocyte injury during this latent window prior to nephron loss. In this review we evaluate the current state of these early-detection modalities, emphasizing their translational potential.

While frequently used synonymously, a nuanced distinction exists between Radiation-Induced Renal Injury (RRI) and Radiation Nephropathy (RN). RRI represents the broad continuum of tissue damage initiated by ionizing radiation, encompassing subclinical molecular events that occur during the latent phase, such as DNA double-strand breaks (gamma-H2AX foci) and oxidative stress. Radiation Nephropathy refers to the definitive clinical syndrome characterized by measurable functional impairment, including hypertension, proteinuria, and a decline in glomerular filtration rate (GFR) (Cohen and Robbins, 2003). Recognizing this distinction is critical for early intervention; while RN describes irreversible structural loss, RRI identifies a pre-fibrotic window where regenerative medicine and novel diagnostics may successfully mitigate disease progression (Table 1).

**TABLE 1:** Nomenclature and clinical distinction in radiation-induced kidney disease.

Domain	Subclinical radiation-induced renal injury (RRI)	Radiation nephropathy (RN)
Definition	Continuum of radiation-mediated renal damage	Clinical syndrome with renal function loss (Cohen and Robbins, 2003)
Primary hallmarks	DNA damage, ROS generation, and pro-inflammatory cytokine release	Symptomatic and measurable organ dysfunction as: hypertension, proteinuria, and progressive azotemia (Cohen and Robbins, 2003).
Stage of detection	Pre-fibrotic/subclinical phase (Ostermann et al., 2020)	Overt/clinical phase (Cohen and Robbins, 2003)
Diagnostic window	Pre-fibrotic/Subclinical: Detected via molecular biomarkers (KIM-1, NGAL) or functional MRI (Tournebize et al., 2025; Brilland et al., 2023)	Clinical/Overt: Detected via standard serum chemistry (Creatinine, BUN) and urinalysis (Cohen and Robbins, 2003; Tran et al., 2014)
Pathological state	Includes reversible tubular stress and early microvascular rarefaction	(Ostermann et al., 2020). Characterized by irreversible glomerulosclerosis and interstitial fibrosis (Cohen and Robbins, 2003; Tran et al., 2014)
Therapeutic implications	Primary target for Regenerative Medicine and cell-free (exosome) interventions (Ostermann et al., 2020; Wang et al., 2026). Represents the end-stage, may require renal replacement therapy	(Cohen and Robbins, 2003; Tran et al., 2014)

Abbreviations: RRI: Radiation-Induced Renal Injury; RN: Radiation Nephropathy; ROS: reactive oxygen species; KIM-1: kidney injury molecule-1; NGAL: neutrophil gelatinase-associated lipocalin; BUN: blood urea nitrogen; MRI: magnetic resonance imaging.

## Diagnostic gap and pathophysiology

Clinical Exposure Contexts: RRI happens after radiotherapy, such as external-beam radiotherapy and conditioning regimens for hematopoietic stem-cell transplantation; accidental or environmental exposures, including industrial incidents, and military or battlefield scenarios involving nuclear detonation or radiological dispersal devices. RRI can also occur after peptide receptor radionuclide therapy such as^177^Lu-DOTATATE, where renal tubular reabsorption and urinary excretion result in disproportionate radiation deposition within the proximal tubules (Kostos et al., 2025). The single fraction X or gamma irradiation doses threshold or its multifaction equivalent to cause RRI is generally 6 Gy or higher (600 cGy or reads).

Known mechanistic features: Exposure of kidneys to sufficient doses of ionizing radiation causes immediate DNA double-strand breaks, triggering cell-cycle arrest, apoptosis, and senescence. Endothelial injury leads to capillary rarefaction and impaired oxygen delivery, while tubular epithelial stress promotes profibrotic signaling through pathways such as TGF-β/SMAD. These events unfold well before reductions in renal filtration become apparent, thereby creating an interval between irradiation and clinical recognition of injury (Verginadis et al., 2025; Li et al., 2018) that may enable timely mitigation. Additionally, although the Renin-Angiotensin-Aldosterone System (RAAS) components including angiotensinogen, renin, and Angiotensin II do not appear to be significantly upregulated in radiation nephropathy, the RAAS system appears to be a key player, given the preventive, mitigating, and therapeutic benefit of RAAS blockade in experimental radiation nephropathy.

But classical cellular inflammation as an excess of white blood cells is absent in radiation nephropathy (Cohen et al., 2020). While other organs like the lungs or the gut show a heavy influx of inflammatory cells after radiation, the kidney does not typically exhibit this “itis' (inflammation) hence the updated use of the word nephropathy instead of nephritis.

## Multimodal early detection of radiation-induced renal injury

**FIGURE 1:**
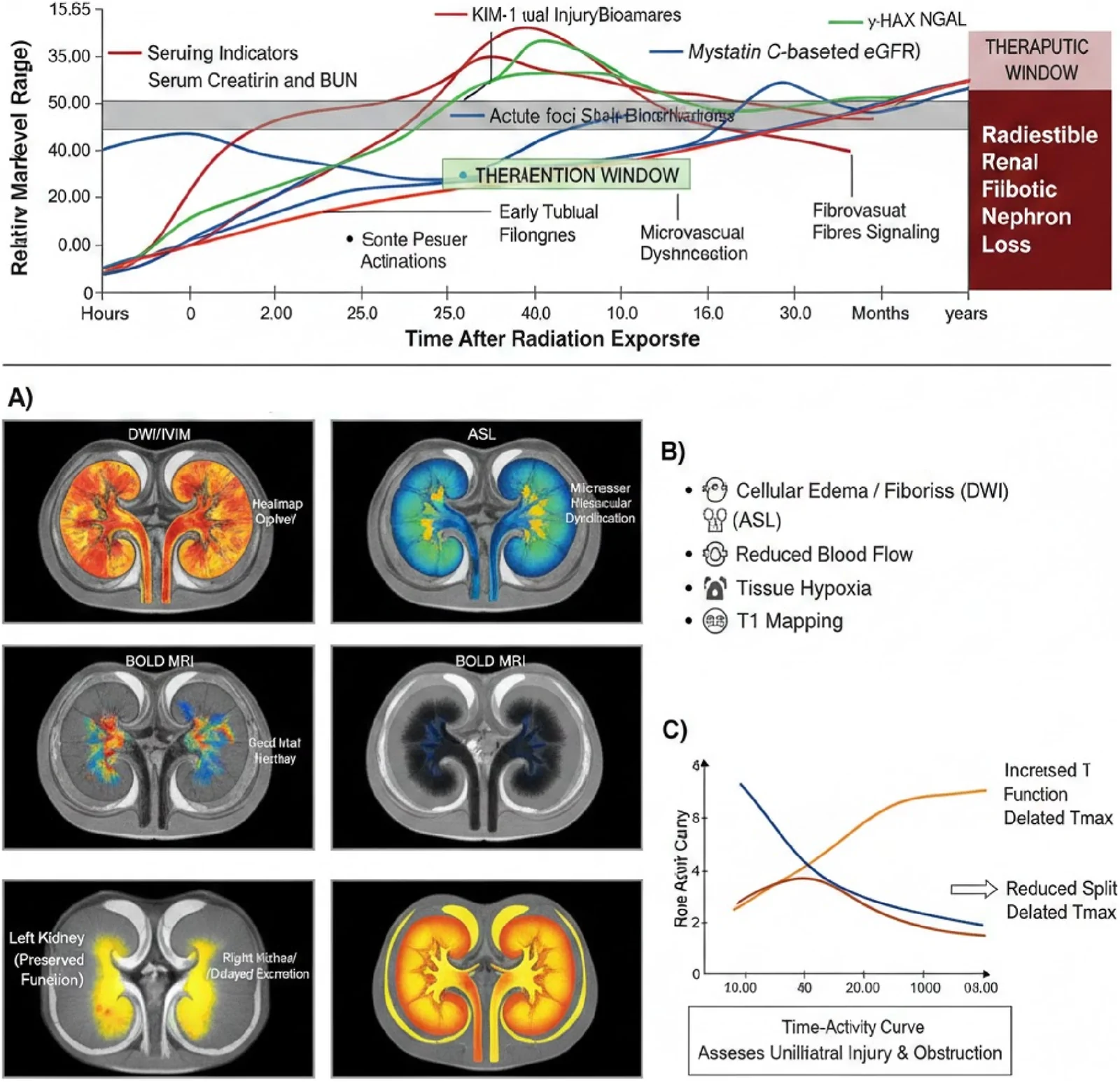
Multimodal Diagnostic Framework for the Early Detection of Radiation-Induced Renal Injury. The transition from lagging functional indicators to pre-fibrotic molecular and imaging signatures defines a critical therapeutic window. **(A)** Non-Contrast Multiparametric MRI (Structural and Physiological) This section focuses on spatial mapping. Unlike traditional scans, these sequences look at the “hidden” physics of the kidney tissue to find damage before the kidney actually stops filtering blood. DWI/IVIM (Diffusion Weighted Imaging): Measures how water molecules move. If water is restricted, it indicates cellular edema (swelling) or early fibrosis (scarring). ASL (Arterial Spin Labeling): A “magnetic” contrast that measures blood flow. It detects microvascular dysfunction—the shrinking of tiny blood vessels that often precedes tissue death. BOLD MRI (Blood Oxygen Level-Dependent): Specifically looks at tissue hypoxia (lack of oxygen). Since radiation damages vessels, the kidney becomes “suffocated,” which triggers the fibrotic signaling (miR-21/miR-29). **(B)** Functional MRI Insights (Diagnostic Markers) This is the interpretive key for the MRI data. It translates the raw images into clinical staging: Cellular Edema: Identifies the immediate structural and fluid-balance response to radiation, characterized by swelling of the renal cells. Unlike typical tissue injury, this process in radiation nephropathy usually occurs independent of a significant inflammatory infiltrate. Reduced Blood Flow: Confirms damage to the peritubular capillaries. T1 Mapping: A specific quantitative tool used to measure interstitial expansion. This is the “holy grail” of pre-fibrotic detection because it can quantify the very first microscopic deposits of collagen. **(C)** Tc-99m MAG3 Renography (Functional and Lateralized) This section addresses “Split Renal Function.” A major problem in urological oncology is that the healthy kidney often works harder to compensate for the irradiated one, keeping the serum creatinine levels looking normal. Time-Activity Curve: Graphs how fast the kidney picks up and clears a radioactive tracer. Delayed Tmax: If the peak of the curve is pushed to the right, it indicates the kidney is struggling to process waste, even if the patient is well. Asymmetry Detection: This is the primary tool for seeing unilateral injury. It proves that one kidney is failing while the other is “masking” the injury, allowing for focal intervention. Proposed Temporal Kinetics of RRI Biomarkers: A schematic timeline illustrating the hypothesized lag between initial radiation exposure and clinical dysfunction. While traditional markers (Serum Creatinine and BUN; grey line) typically remain stable until significant, late-stage nephron loss occurs, research into potential injury-centric biomarkers is ongoing. Current models suggest that tubular markers (KIM-1, NGAL; red/green lines) and genomic damage indicators (gamma-H2AX foci; blue line) may peak during acute or subclinical phases. This suggests a theoretical “Therapeutic Window” where intervention could potentially occur before the onset of irreversible fibrotic nephron loss, though these markers are not yet established for routine clinical use in radiation nephropathy (Bottom Left) Non-Contrast Multiparametric MRI (mpMRI): Emerging functional imaging sequences provide spatially resolved data on renal health. These techniques are being investigated for their ability to assess renal status without the risk of contrast-induced nephropathy, offering a non-invasive tool for monitoring subclinical structural changes. DWI/IVIM: Heatmaps reflecting cellular density and edema, sensitive to early interstitial changes. ASL: Quantitative assessment of microvascular perfusion and blood flow reduction. BOLD MRI: Evaluation of tissue hypoxia through R2* mapping, capturing the transition to a pro-fibrotic microenvironment. T1 Mapping: Detection of early collagen deposition and interstitial expansion (Bottom Right) Tc-99m MAG3 Renography: Time-activity curves illustrating the assessment of “split” renal function. This modality is essential for detecting asymmetrical radiation damage or obstructive uropathy, which may be masked in systemic blood tests by the compensatory function of the contralateral kidney.

Molecular Liquid Biopsy, Functional Imaging, and Translational Surveillance (Figure 1): Radiation-induced renal injury (RRI) progresses along a clinically silent trajectory in which molecular and microstructural damage precedes measurable declines in glomerular filtration. While conventional markers like serum creatinine (SCr) and estimated glomerular filtration rate (eGFR) eventually reflect injury, their utility is limited by their reliance on population-based reference ranges. In an individual patient, SCr may rise significantly from its baseline yet remain within the normal range of the assay, thus masking the early loss of renal function and narrowing the window for timely therapeutic intervention. This diagnostic delay underscores the need for multimodal strategies capable of detecting injury at its earliest biological stages, before irreversible fibrosis develops (Cohen and Robbins, 2003). Early detection could be achieved through complementary modalities: molecular “liquid biopsy” markers of cellular stress, genomic and epigenetic indicators of DNA damage and fibrogenesis, and functional imaging techniques that quantify microstructural and perfusion abnormalities.

## Molecular damage biomarkers (“liquid biopsy”)

### Markers of proximal tubular injury

Biomarkers associated with proximal tubular stress caused by other acute and chronic kidney diseases, such as KIM-1 and NGAL, represent potential candidates for monitoring RRI. While these markers have been validated in non-radiation models of renal injury, their specific utility and kinetic profile in the context of radiation nephropathy remain to be established. Identifying whether these markers can reliably signal early radiation-induced damage, as they do in other nephrotoxic settings, is a primary goal for future clinical use.

Kidney Injury Molecule-1 (KIM-1): Among the most extensively studied candidates are proximal tubular injury markers (KIM-1 and neutrophil gelatinase-associated lipocalin (NGAL) (Virzì et al., 2025). KIM-1 is minimally expressed in healthy kidneys but is robustly upregulated in proximal tubular epithelial cells following injury. It is shed into urine and plasma, where elevations correlate strongly with histologic tubulointerstitial damage and precede changes in serum creatinine. This makes KIM-1 attractive for detecting radiation-associated nephrotoxicity during the latent phase^15^.

Neutrophil Gelatinase-Associated Lipocalin (NGAL): NGAL rises in the bloodstream within hours of tubular stress and has been widely described as a “renal troponin” due to its rapid release. In critical care and cardiac surgery cohorts, serum NGAL identifies potential injury earlier than serum creatinine.

Most evidence supporting KIM-1 and NGAL as early injury markers has been derived from ischemic, toxic, septic, surgical, or chronic kidney disease populations rather than prospective human RRI cohorts. Because RRI is predominantly microvascular, endothelial, and slowly progressive, the magnitude and timing of tubular biomarker release may differ from acute inflammatory or nephrotoxic injury. RRI-specific sampling intervals, diagnostic thresholds, sensitivity, specificity, and incremental predictive value have not been established. These markers should therefore be described as candidate or investigational RRI biomarkers rather than clinically validated diagnostic tests.

While direct data specific to Radiation-Induced Renal Injury (RRI) are currently limited, extrapolation from high-dose radiation and combined-injury models supports the potential role of both KIM-1 and NGAL as useful biomarkers for detecting acute tubular damage following radiation exposure (Virzì et al., 2025).

### Clinical implication: combined markers

The integration of injury and functional biomarkers represents a rational approach to improving early detection of RRI. While tubular injury markers such as KIM-1 and NGAL reflect early cellular stress, filtration markers such as serum creatinine or cystatin C capture downstream functional decline. Combining these markers may improve sensitivity for detecting subclinical injury, enabling earlier identification of patients at risk for progression. This multimodal biomarker approach may also provide a framework for risk stratification and guide the timing of advanced imaging or therapeutic interventions (Ostermann et al., 2020).

### Genomic and epigenetic indicators

Phosphorylated histone H2AX (γ-H2AX) rapidly localizes to DNA double-strand break sites within minutes of radiation exposure. Persistence of γ-H2AX foci beyond 24 h reflects impaired DNA repair and predicts downstream tissue injury and fibrosis (Kunwar and Haston, 2015; Zhang et al., 2025; Solana-Peña et al., 2025). Advances in automated scoring systems now permit scalable biodosimetry and dose estimation based on γ-H2AX, potentially valuable for triage following mass radiation exposure (Raavi et al., 2021; Georgoulis et al., 2017).

### MicroRNAs: drivers of the pre-fibrotic transition

Circulating and urinary microRNAs do more than indicate established scarring; they reflect early and dynamic changes in cellular signaling that may occur before visible tissue damage. Instead of representing permanent injury, these microRNAs reveal the molecular “tipping point' at which acute damage starts to evolve into chronic fibrosis (Loboda et al., 2016; Glowacki et al., 2013). miR-21 (The Pathogenic Signal): miR-21 functions as an early driver of fibroblast activation. Its increase during the pre-fibrotic phase signals the start of extracellular matrix deposition, occurring before changes are detectable by traditional imaging or filtration markers (Glowacki et al., 2013; Bhayana et al., 2017). miR-29 Family (The Lost Protectors): Conversely, the miR-29 family normally functions to suppress collagen gene programs. Its early downregulation following radiation exposure serves as a molecular “red flag,' indicating the loss of anti-fibrotic defenses and a high risk of subsequent progression to organ scarring (Qin et al., 2011).

The ratio of these signaling molecules (Pat et al., 2012) may identify the latent transition from injury to fibrosis, offering a window for intervention before the damage becomes irreversible.

### Advanced functional imaging

Multiparametric MRI (Non-Contrast Preferred) provides complementary insights into microstructure, perfusion, and oxygenation.

Functional MRI sequences offer a noninvasive means to monitor the progression from acute radiation-induced injury to chronic kidney disease. Among these, diffusion-weighted imaging (DWI) and intravoxel incoherent motion (IVIM) are especially valuable. They detect reductions in cortical apparent diffusion coefficient (ADC) and perfusion fraction, which indicate early microvascular congestion and cellular swelling. These changes precede significant collagen buildup and reveal early disturbances in tissue fluid balance and capillary integrity (Jerome et al., 2021; Caroli et al., 2018).

Arterial spin labeling (ASL) complements this by quantitatively measuring renal cortical blood flow without contrast agents. Decreased ASL perfusion signals early microvascular rarefaction, a critical factor in the pre-fibrotic stage, making it a potential early marker for progression toward irreversible damage (Mao et al., 2023; Odudu et al., 2018). Additionally, T1 mapping and magnetization transfer imaging highlight interstitial alterations; increases in T1 relaxation times and reduced corticomedullary differentiation correspond to interstitial edema and early extracellular matrix remodeling, occurring before fibrosis is evident histologically (Wolf et al., 2018).

Blood oxygen level–dependent (BOLD) MRI further contributes by assessing tissue oxygenation via R2* values. Since hypoxia is central to the link between initial radiation injury and fibrosis, BOLD imaging provides real-time insights into metabolic stress within the kidney tissue. While dynamic contrast-enhanced MRI can offer more functional data, its use is limited by nephrotoxicity concerns, though safer contrast agents may broaden its future use (Pruijm et al., 2018; Sugiyama et al., 2020).

Dynamic contrast-enhanced MRI offers additional functional data but is currently limited by nephrotoxicity concerns; emerging non-nephrotoxic contrast agents may expand its role (Zhang et al., 2025; Solana-Peña et al., 2025; Raavi et al., 2021; Georgoulis et al., 2017).

Although DWI/IVIM and ASL are established tools for detecting early renal microstructural and perfusion changes, their specific role in radiation-induced kidney injury requires further validation. A key limitation is their sensitivity but lack of specificity, as similar imaging changes can result from aging or existing chronic kidney disease. This overlap highlights the importance of combining imaging findings with molecular and clinical data to improve diagnostic accuracy in assessing radiation nephropathy (Liu et al., 2017).

Dynamic contrast-enhanced MRI offers additional functional data but is currently limited by nephrotoxicity concerns; emerging non-nephrotoxic contrast agents may expand its role (Zhang et al., 2025; Solana-Peña et al., 2025; Raavi et al., 2021; Georgoulis et al., 2017).

We recognize that these MRI techniques would be sensitive but not specific indicators. They reflect changes in tissue diffusion and blood flow parameters that are also affected by aging and pre-existing chronic kidney disease. This confounds the imaging patterns expected in radiation nephropathy (Tournebize et al., 2025; Rabadi et al., 2025; Stabinska et al., 2025).

Consequently, the role of functional MRI in RRI research should be approached with caution. To establish these as reliable indicators, future studies must validate RRI-specific signatures that can be differentiated from age- or disease-related decline. Imaging data then can be integrated to clinical context and the specific molecular/urinary biomarkers discussed previously to improve diagnostic specificity (Bjornstad et al., 2025).

## Confounding comorbidities and attribution of MRI abnormalities

Multiparametric MRI cannot diagnose RRI as a standalone modality. Reduced perfusion, altered diffusion, impaired oxygenation, T1 prolongation, and loss of corticomedullary differentiation may also occur with aging, hypertension, diabetes, vascular disease, baseline CKD, anemia, dehydration, chemotherapy-associated nephrotoxicity, or other renal disorders. Radiation attribution therefore requires integration of imaging findings with exposure timing, patient-specific renal dosimetry, baseline renal status, clinical confounders, and longitudinal biomarker data.

Several strategies may improve attribution. Pre-treatment MRI can establish an individual baseline, while longitudinal delta measurements can distinguish new post-exposure changes from stable chronic abnormalities. When renal irradiation is markedly asymmetric, the lower-dose kidney may provide a partial within-patient comparator, although systemic disease and scatter dose prevent it from functioning as a perfect control. Spatial registration of MRI abnormalities with three-dimensional renal dose maps may also strengthen causal inference. Future studies should use matched control groups, prespecified adjustment for diabetes, hypertension and CKD, and harmonized imaging protocols across scanner platforms.

Ultrasound Shear-Wave Elastography: Shear-wave elastography provides a non-invasive estimate of tissue stiffness, but measurements are affected by hydration, renal perfusion, blood pressure, respiratory motion, probe pressure, acquisition depth, region-of-interest placement, scanner platform, and tissue anisotropy. No universal RRI-specific stiffness threshold should be proposed without direct validation. RRI studies should instead standardize acquisition procedures and evaluate longitudinal within-patient changes alongside functional, molecular, and dosimetric data (Cao et al., 2023; Olson et al., 2023).

Nuclear Medicine: Tc-99m MAG3 Renography: Tc-99m MAG3 renography should be interpreted using standardized acquisition and reporting procedures. Relevant parameters include relative or differential renal function, time to peak activity, renogram curve morphology, post-diuretic clearance, residual tracer activity, drainage indices, and technical adequacy of the examination. No single parameter should be interpreted in isolation. MAG3 can identify functional asymmetry and impaired drainage, but these findings are not specific to radiation injury and must be evaluated with anatomical imaging, dosimetry, and the clinical context.

### Nuclear medicine and standardized renal imaging

Established guidelines from the Society of Nuclear Medicine and Molecular Imaging (SNMMI) and the European Association of Nuclear Medicine (EANM) ensure reproducible acquisition and interpretation of renal scintigraphy.

Adhering to these international standards is particularly valuable in the context of radiation injury for several reasons: i). Precision in Laterality: These protocols provide a reliable baseline and longitudinal tracking of differential renal function, which is critical when radiation fields involve only one kidney or involve asymmetrical dosing; ii). Assessment of Obstruction: Standardized diuretic renography (e.g., MAG3 scans) helps distinguish between true functional decline and mechanical obstruction, a common diagnostic dilemma in pelvic or abdominal radiation cases; iii). Inter-Institutional Consistency: Using SNMMI/EANM-validated methods allows for the comparison of data across different clinical trials and centers, facilitating the multicenter research needed to better define RRI (Peters, 2025; Taylor et al., 2018).

## Special exposure contexts

Certain clinical settings place patients at particularly high risk for RRI and therefore warrant proactive surveillance. Chronic radiation nephropathy is well documented following total body irradiation in hematopoietic cell transplantation, and renin–angiotensin–aldosterone system blockade has demonstrated mitigative benefit in animal models, underscoring the importance of timely detection (Cohen et al., 2020). Similarly, targeted radionuclide therapies, including peptide receptor radionuclide therapy (^177^Lu-DOTATATE), expose the proximal tubules to potentially harmful radiation doses due to tubular reabsorption of the isotope. Although long-term outcomes are often acceptable with protective protocols, chronic renal failure can result, which emphasizes the role of personalized dosimetry and early biomarker-guided monitoring (Kostos et al., 2025).

The presence of pre-existing chronic kidney disease may predispose to renal radiation injury. This has been suggested in the case of radionuclide injury (Rodríguez-Ribera et al., 2015). Future studies must address this concern, given the population prevalence of chronic kidney disease. Models of radiation injury should be created on a background of CKD to identify this predisposition and also whether mitigators retain their benefit in those models.

## Practical monitoring and research framework

Following radiation exposure, monitoring strategies diverge between established clinical care and the investigational techniques used in laboratory models or clinical trials (Figure 1).

1. Baseline Assessment
  - Clinical (Human): Establish baseline creatinine- and cystatin C–based eGFR and proteinuria. Document comorbidities such as hypertension and diabetes and current nephrotoxin use.
  - Investigational: In high-risk cohorts or research models, consider baseline KIM-1, NGAL, and multiparametric MRI to establish a biological “fingerprint' prior to potential injury.
2. Early Post-Exposure (The Latent Phase)
  - Clinical (Human): do serial eGFR and albuminuria monitoring.
  - Investigational: In cases of uncertain dose or combined injury, explore gamma-H2AX biodosimetry or assay for NGAL/KIM-1 to detect early tubular injury. Non-contrast functional MRI may be considered in research settings if subclinical abnormalities are suspected.
3. Longitudinal Surveillance
  - Clinical (Human): Management follows standard Chronic Kidney Disease (CKD) guidelines, focusing on blood pressure control (ACEi/ARBs) and metabolic stability.
  - Investigational: Serial biomarker collection (every 1–3 months) and repeat MRI imaging to correlate early signaling shifts with eventual functional decline. MAG3 renography (per SNMMI/EANM guidelines) is indicated if structural or functional asymmetry is suspected.

### Proposed decision triggers (hypothetical)

The following points represent a theoretical framework for future clinical trials rather than current established medical practice.

- Subclinical Stress: If biomarker elevation occurs without eGFR decline, research protocols might suggest intensifying nephroprotection and shortening the surveillance interval.
- Physiological Shifts: Imaging evidence of reduced perfusion or diffusion could serve as a potential trigger for evaluating investigational antifibrotic therapies or other trial-based interventions before irreversible scarring occurs.

## A novel non-invasive diagnostic path using urinary extracellular vesicles

The progression from the latent phase of radiation-induced renal injury (RRI) to irreversible fibrosis is apt to be associated with significant events in renal molecular signaling. Urinary extracellular vesicles (uEVs) could provide a non-invasive platform for assessing these intra-renal changes, as they encapsulate and protect bioactive cargo including microRNAs (miRNAs) and messenger RNAs (mRNAs) from degradation within the urinary milieu. Because uEVs originate from renal epithelial cells, their molecular content reflects active cellular processes occurring within those cells. Among emerging candidates, the miR-30 family and TGF-β mRNA have been identified as potential early biomarkers of radiation-associated injury (Medeiros et al., 2020; Petzuch et al., 2022; Chung and Lan, 2015; Wang et al., 2012; Tepus et al., 2022) (Table 2).

**TABLE 2:** Molecular signatures and biomarkers of early radiation-induced renal injury.

Modality/Tool	Biological source	Primary focus	Earliest detectable signal	Clinical/Research utility
KIM-1 (Brilland et al., 2023)	Urine/Plasma	Proximal tubular epithelial injury	Early (pre- creatinine)	Specific marker of tubular damage; strong translational and human validation data
NGAL (Virzì et al., 2025)	Urine/Plasma	Tubular stress and injury	Hours–days	Rapid early alert biomarker; rises before serum creatinine
Cystatin C (Estrella et al., 2025; Carrero et al., 2023)	Plasma	Glomerular filtration function	Earlier than creatinine	Improves early risk stratification when combined with tubular markers*
γ-H2AX foci (Raavi et al., 2021; Georgoulis et al., 2017)	Peripheral blood cells/tissue	DNA double-strand breaks (DSBs)	Minutes–hours	Biodosimetry tool; useful for dose estimation and acute radiation triage
Multiparametric MRI (DWI/IVIM, ASL, BOLD)	Imaging	Renal microstructure, perfusion, oxygenation	Pre-fibrotic structural changes	Sensitive to early microvascular and fibrotic alterations; non-contrast protocols feasible
Shear wave Elastography (SWE) (Cao et al., 2023)	Ultrasound	Tissue stiffness (fibrosis surrogate)	Early stiffness alterations	Non-invasive fibrosis assessment; operator-dependent
99mTc-MAG3 Renography (Peters, 2025; Taylor et al., 2018)	Nuclear imaging	Split renal function and drainage	Functional asymmetry	Standardized acquisition; useful for detecting differential renal impairment

Abbreviations: KIM-1: kidney injury molecule-1, NGAL: neutrophil gelatinase-associated lipocalin, γ-H2AX: phosphorylated histone H2AX, DWI: diffusion-weighted imaging, IVIM: intravoxel incoherent motion, ASL: arterial spin labeling, BOLD: blood oxygen level–dependent imaging, SWE: shear wave elastography, 99mTc-MAG3: technetium-99m mercaptoacetyltriglycine. * Provides incremental statistical gain in risk stratification when combined with tubular markers, though its independent clinical value and specific utility in radiation nephropathy remain unvalidated’. Uses SNMMI/EANM standards for reproducible detection of differential renal impairment; historical data confirm that early perfusion abnormalities correlate with subsequent loss of GFR.

### The miR-30 family and podocyte integrity

Members of the miR-30 family are highly expressed in healthy podocytes and play a critical role in maintaining cytoskeletal organization and glomerular filtration barrier stability. In experimental models of renal injury, oxidative stress has been associated with downregulation of uEV-associated miR-30 (Liu et al., 2016). Podocyte injury has been implicated in a murine model of radiation nephropathy (Ahmad et al., 2023) and we have shown early evidence of podocyte injury by scanning electron microscopy in a rat model (Sharma et al., 2008)). Reductions in miR-30 levels may precede the development of overt albuminuria, suggesting utility as an early indicator of podocyte stress, detachment, or apoptosis. Mechanistically, loss of miR-30 permits upregulation of pro-apoptotic mediators such as p53 and contributes to activation of signaling pathways including Notch, which promote epithelial–mesenchymal transition (EMT) of podocytes and accelerate tubulointerstitial fibrosis (Medeiros et al., 2020; Petzuch et al., 2022; Chung and Lan, 2015; Wang et al., 2012; Tepus et al., 2022).

### TGF-β mRNA and pro-fibrotic signaling

Transforming growth factor-β (TGF-β) is a central regulator of renal fibrogenesis. While circulating TGF-β protein levels may lack specificity, the detection of TGF-β mRNA within uEVs more directly reflects intrarenal transcriptional activation of pro-fibrotic pathways. Elevated uEV-associated TGF-β mRNA has been linked to activation of myofibroblasts and extracellular matrix deposition, processes that drive irreversible structural remodeling. Moreover, increased TGF-β signaling frequently coincides with upregulation of pro-fibrotic microRNAs such as miR-21, which suppress anti-fibrotic regulators and amplify tissue remodeling cascades, ultimately contributing to progression toward chronic kidney disease (Meng et al., 2015).

## Clinical utility and integration

The clinical power of these markers lies in their kinetic profile. Unlike a single renal biopsy, uEV signatures can be monitored longitudinally post-irradiation. A signature characterized by decreasing miR-30 and increasing TGFβ mRNA could provide a high-fidelity risk profile for radiation nephropathy, potentially identifying patients who would benefit from adroitly-timed intervention with ACE inhibitors, experimental TGFβ antagonists, or as-yet-to-be-discovered mitigators before eGFR begins its decline (Meng et al., 2015).

In an integrated diagnostic workflow, a decreasing miR-30 signal combined with an elevated TGF-β mRNA signature would constitute a “molecular alert.' This signature could identify an irradiated subject in the “pre-fibrotic' window, allowing precisely-timed mitigative therapies such as ACE inhibitors or TGF-β antagonists before the irreversible rise of serum creatinine occurs (Lenarczyk et al., 2009). Addressing the aforementioned technical disadvantages through automated, microfluidic-based isolation platforms will be the next critical step in bringing this “liquid biopsy' to the clinic.

Together, we suggest that renal injury following radiation exposure is modifiable when detected early which provides a strong rationale for implementing systematic surveillance strategies. The following table compares traditional lagging indices with the emerging “liquid biopsy' approach using urinary extracellular vesicles (uEVs) (Table 3).

**TABLE 3:** Comparative diagnostic characteristics of conventional and molecular markers relevant to radiation-induced renal injury.

Feature	sCr (Estrella et al., 2025; Carrero et al., 2023; Dewit et al., 1990)	CysC (Estrella et al., 2025; Carrero et al., 2023; Carrero et al., 2023)	uEV miR-30 Family (Meng et al., 2015)	uEV TGF-β mRNA (Meng et al., 2015)
Primary Utility	Estimation of glomerular filtration rate (GFR)	Earlier estimation of GFR decline*	Indicator of podocyte stress and structural integrity	Indicator of intrarenal pro-fibrotic transcriptional activity
Sensitivity to Early Injury	Low; may rise only after substantial nephron loss	Moderate; detects earlier GFR decline	Potentially high; may detect pre-clinical podocyte stress	Potentially high; reflects activation of fibrogenic pathways
Specificity	Limited; influenced by muscle mass, diet, age	Moderate; influenced by inflammation and thyroid status	Relative specificity for podocyte-derived injury	Reflects renal fibrotic signaling but not radiation-specific
Temporal Response	Delayed	May be earlier than sCr	Early molecular alteration (days to weeks post-exposure in experimental models)	May precede overt fibrosis and functional decline
Biological Mechanism	End product of muscle creatine metabolism	Ubiquitous cysteine protease inhibitor produced by nucleated cells	Regulatory microRNA involved in cytoskeletal stability and Notch/p53 modulation	mRNA reflecting TGF-β pathway activation driving myofibroblast differentiation and ECM deposition
Clinical Role in RRI	Monitoring established renal dysfunction	Detection and risk stratification	Identification of early structural injury	Monitoring transition toward fibrotic remodeling

Abbreviations: sCr: serum creatinine, CysC: cystatin C, uEV: urinary extracellular vesicle, ECM: extracellular matrix, TGF-β: transforming growth factor-beta.* Facilitates slightly earlier detection of filtration decline in general cohorts, though its specific clinical value and performance in radiation-induced injury remain unproven. While cystatin C is often cited for its potential to detect filtration decline earlier than serum creatinine, this lead time remains clinically unproven and potentially over-emphasized in the context of radiation nephropathy. Its utility in cancer patients is particularly problematic, as systemic inflammation and certain malignancies can alter cystatin C levels independent of glomerular filtration, complicating its reliability as a standalone marker for radiation-induced injury.

## Key advantages of uEV signatures

**FIGURE 2:**
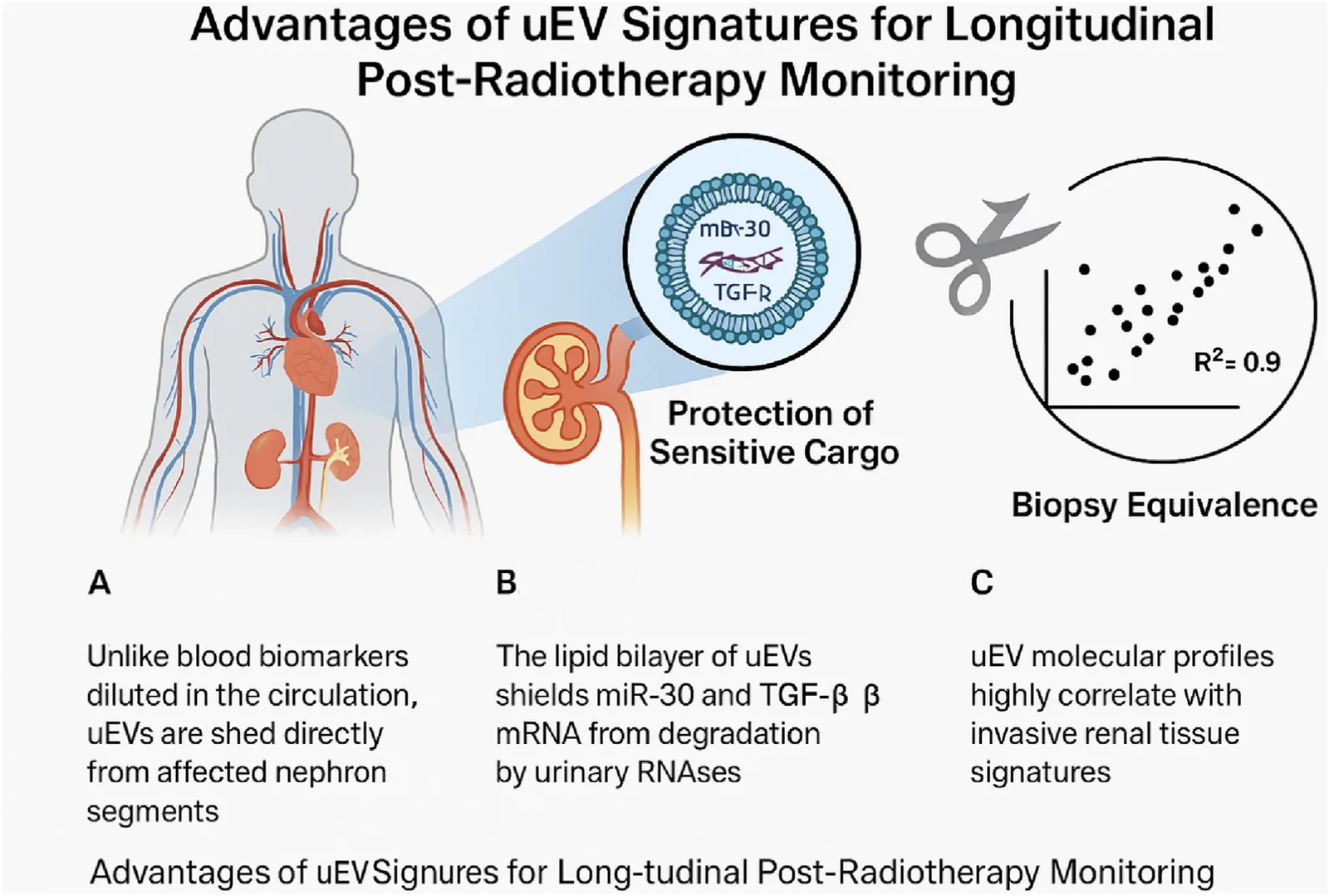
Comparison of Urinary Extracellular Vesicles (uEVs) and Systemic Blood Biomarkers for the Surveillance of Radiation-Induced Renal Damage. The schematic highlights three pivotal advantages of uEVs in precision monitoring following radiation exposure. **(A)** Localized Signal (Direct Shedding): Unlike serum biomarkers that originate from diverse tissues and are diluted within the large systemic blood volume, uEVs are shed directly from specific nephron segments (e.g., podocytes, proximal tubule epithelial cells) into the ultrafiltrate. This provides a “pure,” highly concentrated molecular readout of the renal microenvironment without systemic “noise.” **(B)** Protection of Sensitive Cargo (Lipid Bilayer Integrity): The robust lipid bilayer of the EV acts as a protective shield for labile genetic material, such as miR-30 and TGF-β mRNA. This encapsulation prevents degradation by high concentrations of urinary RNases, ensuring the stability of the molecular signature and allowing for reproducible longitudinal measurements. **(C)** may correlate with selected biopsy-derived molecular findings (Molecular Correlation): uEV molecular profiles serve as a “liquid biopsy. Evidence indicates that the proteomic and transcriptomic signatures within uEVs closely mirror the molecular alterations found in invasive renal tissue samples. This correlation establishes uEVs as a viable, non-invasive alternative for frequent serial monitoring, potentially eliminating the need for invasive needle biopsies in post-RT patients.

- Unlike blood-based biomarkers, urinary extracellular vesicles (uEVs) offer several distinct advantages for longitudinal monitoring of radiation-induced renal injury. Because uEVs are directly shed from nephron segments into the urine, they provide a enriched for urinary-tract and renal signals of renal cellular activity, avoiding the dilution and systemic confounding factors common with circulating biomarkers. Additionally, the lipid bilayer of extracellular vesicles protects their molecular cargo, including microRNAs like miR-30 and mRNA transcripts such as TGF-β—from degradation by urinary RNases, ensuring greater stability and consistency of measurements over time. Importantly, growing evidence shows that uEV molecular profiles closely mirror findings from invasive renal biopsies, supporting their potential as a non-invasive “liquid biopsy” for assessing renal injury and reducing the need for repeated tissue sampling during post-radiotherapy surveillance (Wu et al., 2021; Grange et al., 2023; Street et al., 2017; Grange et al., 2023) (Figure 2).

## Current challenges and disadvantages

While uEVs offer a paradigm shift in renal diagnostics, several technical and translational hurdles must be addressed before they can be integrated into routine clinical practice for RRI monitoring. The clinical adoption of uEVs for RRI diagnosis faces several significant limitations:

1. Lack of Standardization in Isolation: There is currently no “gold standard' for uEV extraction. Techniques vary from ultracentrifugation (labor-intensive and low yield) to size-exclusion chromatography and polymer-based precipitation, each resulting in different vesicle purity and concentration. This heterogeneity complicates the comparison of results.
2. Normalization Challenges in uEV Analysis

Urinary creatinine remains the standard for normalization of any substance in the urine to ensure reproducible data across different collection time points. But the relative change value for urine creatinine levels introduces some variability that affects the precision of measurement (PMID 15222627).

3. High Technical Cost and Complexity: Real-time analysis of miRNA and mRNA cargo requires specialized equipment (e.g., droplet digital PCR or Nanostring) and advanced bioinformatics expertise, which may not be available in many centers; and
4. Contamination with Non-Renal EVs: Urine can contain EVs from the bladder, prostate, or systemic circulation (leaked through a damaged glomerular barrier). Distinguishing nephron-specific signals from these “noise' sources requires stringent immuno-affinity capture of tissue-specific markers ^43-^ (Meng et al., 2015).
5. Much of the available evidence for clinical use of EVs remains derived from small, single-center cohorts or preclinical models, with relatively few prospective human studies specifically focused on RRI.
6. Biomarker clinical performance may be confounded by comorbid conditions common in oncology and transplant populations, including baseline chronic kidney disease, systemic inflammation, and nephrotoxic exposures, which can reduce specificity of any marker of radiation-related injury.

## Future directions: AI-enabled integrated diagnostics

Consensus statements for many conditions or injuries often advocate for multi-marker panels that integrate injury, stress, and functional indicators. A transformative frontier in this space is the application of liquid biopsy specifically the analysis of exfoliated cells and extracellular vesicles (EVs) in the urine. Unlike traditional plasma markers, urinary EVs provide a highly localized molecular snapshot of the renal parenchyma, carrying proteomic and microRNA (miRNA) signatures that reflect active tubular stress and early fibrotic signaling (Meng et al., 2015).

The integration of these high-dimensional “liquid' data points into Machine Learning (ML) frameworks holds significant promise. By combining longitudinal urinary biomarker trajectories with multiparametric MRI (mpMRI) — such as BOLD and Diffusion-Weighted Imaging (DWI) AI-driven models may soon enable:

1. Individualized RRI Risk Scores to stratify patients based on pre-treatment molecular profiles and early post-exposure changes.
2. Optimized Surveillance Intervals that enable shifting from standardized follow-up to “dynamic monitoring' based on real-time biological shifts; and
3. Precision Mitigative Therapy: Guiding the administration of ACE inhibitors or as-yet-to-be-discovered other mitigators before onset of tissue injury.

## Conclusion

We suggest that surveillance for radiation-induced renal injury should begin before measurable declines in glomerular filtration occur. Incorporating tubular injury biomarkers such as KIM-1 or NGAL alongside accurate renal functional estimates, followed by targeted functional imaging when abnormalities emerge, provides a rational and clinically actionable framework for identifying subjects during the latent, potentially reversible phase of injury. Such an approach enables enrollment into preventive, mitigating or antifibrotic trials. By shifting detection from late functional decline to early molecular and microstructural changes, multimodal monitoring has the potential to transform RRI management from reactive damage control to proactive preservation of renal function.

## References

[B1] Ahmad A. Shi J. Ansari S. Merscher S. Pollack A. Zeidan Y. (2023). Radiation nephropathy: mechanisms of injury and recovery in a murine model. Radiother. Oncol. 187, 109813. 10.1016/j.radonc.2023.109813 37468066 PMC11648365

[B2] Bhayana S. Song F. Jacob J. Fadda P. Denko N. C. Xu-Welliver M. (2017). Urinary miRNAs as biomarkers for noninvasive evaluation of radiation-induced renal tubular injury. Radiat. Res. 188, 626–635. 10.1667/RR14828.1 28977780 PMC7263377

[B3] Bjornstad P. Narongkiatikhun P. Benzing T. Fuhrman D. Y. Inker L. Kestenbaum B. (2025). A comprehensive framework for kidney function assessment: summary of the national institute of diabetes and digestive and kidney diseases reimagining kidney function assessment workshop. Kidney Int. Rep. 10, 2919–2936. 10.1016/j.ekir.2025.06.040 40980679 PMC12446987

[B4] Brilland B. Boud'hors C. Wacrenier S. Blanchard S. Cayon J. Blanchet O. (2023). Kidney injury molecule 1 (KIM-1): a potential biomarker of acute kidney injury and tubulointerstitial injury in patients with ANCA-glomerulonephritis. Clin. Kidney J. 16, 1521–1533. 10.1093/ckj/sfad071 37664565 PMC10468750

[B5] Cao H. Ke B. Lin F. Xue Y. Fang X. (2023). Shear wave elastography for assessment of biopsy-proven renal fibrosis: a systematic review and meta-analysis. Ultrasound Med. Biol. 49, 1037–1048. 10.1016/j.ultrasmedbio.2023.01.003 36746743

[B6] Caroli A. Schneider M. Friedli I. Ljimani A. De Seigneux S. Boor P. (2018). Diffusion-weighted magnetic resonance imaging to assess diffuse renal pathology: a systematic review and statement paper. Nephrol. Dial. Transpl. 33, ii29–ii40. 10.1093/ndt/gfy163 30137580 PMC6106641

[B7] Carrero J. J. Fu E. L. Sang Y. Ballew S. Evans M. Elinder C. G. (2023). Discordances between Creatinine- and cystatin C-Based estimated GFR and adverse clinical outcomes in routine clinical practice. Am. J. Kidney Dis. 82, 534–542. 10.1053/j.ajkd.2023.04.002 37354936

[B8] Chung A. C. Lan H. Y. (2015). MicroRNAs in renal fibrosis. Front. Physiol. 6, 50. 10.3389/fphys.2015.00050 25750628 PMC4335396

[B9] Cohen E. P. Robbins M. E. (2003). Radiation nephropathy. Semin. Nephrol. 23, 486–499. 10.1016/s0270-9295(03)00093-7 13680538

[B10] Cohen E. P. Robbins M. E. Whitehouse E. Hopewell J. W. (1997). Stenosis of the tubular neck: a possible mechanism for progressive renal failure. J. Lab. Clin. Med. 129, 567–573. 10.1016/s0022-2143(97)90011-1 9142053

[B11] Cohen E. P. Farese A. M. Parker G. A. Kane M. A. MacVittie T. J. (2020). Lack of cellular inflammation in a non-human primate model of radiation nephropathy. Health Phys. 119, 588–593. 10.1097/HP.0000000000001329 32941291 PMC8932376

[B12] Dawson L. A. Kavanagh B. D. Paulino A. C. Das S. K. Miften M. Li X. A. (2010). Radiation-associated kidney injury. Int. J. Radiat. Oncol. Biol. Phys. 76, S108–S115. 10.1016/j.ijrobp.2009.02.089 20171504

[B13] Dewit L. Anninga J. K. Hoefnagel C. A. Nooijen W. J. (1990). Radiation injury in the human kidney: a prospective analysis using specific scintigraphic and biochemical endpoints. Int. J. Radiat. Oncol. Biol. Phys. 19, 977–983. 10.1016/0360-3016(90)90022-c 1976615

[B14] Estrella M. M. Ballew S. H. Sang Y. Grams M. E. Coresh J. Surapaneni A. (2025). Discordance in Creatinine- and cystatin C-Based eGFR and clinical outcomes: a meta-analysis. Jama 334, 1915–1926. 10.1001/jama.2025.17578 41202182 PMC12595547

[B15] Georgoulis A. Vorgias C. E. Chrousos G. P. Rogakou E. P. (2017). Genome instability and γH2AX. Int. J. Mol. Sci. 18. 10.3390/ijms18091979 28914798 PMC5618628

[B16] Glowacki F. Savary G. Gnemmi V. Buob D. Van der Hauwaert C. Lo-Guidice J. M. (2013). Increased circulating miR-21 levels are associated with kidney fibrosis. PLoS One 8, e58014. 10.1371/journal.pone.0058014 23469132 PMC3585177

[B17] Grange C. Dalmasso A. Cortez J. J. Spokeviciute B. Bussolati B. (2023). Exploring the role of urinary extracellular vesicles in kidney physiology, aging, and disease progression. Am. J. Physiol. Cell Physiol. 325, C1439–c1450. 10.1152/ajpcell.00349.2023 37842748 PMC10861146

[B18] Harb A. H. Abou Fadel C. Sharara A. I. (2014). Radiation enteritis. Curr. Gastroenterol. Rep. 16, 383. 10.1007/s11894-014-0383-3 24604730

[B19] Jerome N. P. Caroli A. Ljimani A. (2021). Renal diffusion-weighted imaging (DWI) for apparent diffusion coefficient (ADC), intravoxel incoherent motion (IVIM), and diffusion tensor imaging (DTI): basic concepts. Methods Mol. Biol. 2216, 187–204. 10.1007/978-1-0716-0978-1_11 33476001 PMC9703200

[B20] Kal H. B. van Kempen-Harteveld M. L. (2006). Renal dysfunction after total body irradiation: dose-effect relationship. Int. J. Radiat. Oncol. Biol. Phys. 65 (4), 1228–1232. 10.1016/j.ijrobp.2006.02.021 16682132

[B21] Klaus R. Niyazi M. Lange-Sperandio B. (2021). Radiation-induced kidney toxicity: molecular and cellular pathogenesis. Radiat. Oncol. 16, 43. 10.1186/s13014-021-01764-y 33632272 PMC7905925

[B22] Kostos L. Buteau J. P. Xie J. Cardin A. Akhurst T. Alipour R. (2025). Lutetium-177 [^177^Lu]Lu-PSMA-I&T plus radium-223 in patients with metastatic castration-resistant prostate cancer (AlphaBet): an interim analysis of the investigator-initiated, single-centre, single-arm, phase 1/2 trial. Lancet Oncol. 26 (11), 1479–1488. 10.1016/S1470-2045(25)00559-5 41119954

[B23] Kunwar A. Haston C. K. (2015). DNA damage at respiratory distress, but not acute time-points, correlates with tissue fibrosis following thoracic radiation exposure in mice. Int. J. Radiat. Biol. 91, 360–367. 10.3109/09553002.2015.997897 25529973

[B24] Lenarczyk M. Cohen E. P. Fish B. L. Irving A. A. Sharma M. Driscoll C. D. (2009). Chronic oxidative stress as a mechanism for radiation nephropathy. Radiat. Res. 171, 164–172. 10.1667/RR1454.1 19267541 PMC2664164

[B25] Li M. You L. Xue J. Lu Y. (2018). Ionizing radiation-induced cellular senescence in normal, non-transformed cells and the involved DNA damage response: a mini review. Front. Pharmacol. 9, 522. 10.3389/fphar.2018.00522 29872395 PMC5972185

[B26] Liu L. Lin W. Zhang Q. Cao W. Liu Z. (2016). TGF-β induces miR-30d down-regulation and podocyte injury through Smad2/3 and HDAC3-associated transcriptional repression. J. Mol. Med. Berl. 94, 291–300. 10.1007/s00109-015-1340-9 26432290

[B27] Liu Z.-C. Yan L.-F. Hu Y.-C. Sun Y.-Z. Tian Q. Nan H.-Y. (2017). Combination of IVIM-DWI and 3D-ASL for differentiating true progression from pseudoprogression of glioblastoma multiforme after concurrent chemoradiotherapy: study protocol of a prospective diagnostic trial. BMC Med. Imaging 17, 10. 10.1186/s12880-017-0183-y 28143434 PMC5286785

[B28] Loboda A. Sobczak M. Jozkowicz A. Dulak J. (2016). TGF-β1/Smads and miR-21 in renal fibrosis and inflammation. Mediat. Inflamm. 2016, 8319283. 10.1155/2016/8319283 27610006 PMC5005604

[B29] Mao W. Ding Y. Ding X. Fu C. Cao B. Kuehn B. (2023). Capability of arterial spin labeling and intravoxel incoherent motion diffusion-weighted imaging to detect early kidney injury in chronic kidney disease. Eur. Radiol. 33, 3286–3294. 10.1007/s00330-022-09331-z 36512040

[B30] Medeiros T. Myette R. L. Almeida J. R. Silva A. A. Burger D. (2020). Extracellular vesicles: cell-derived biomarkers of glomerular and tubular injury. Cell Physiol. Biochem. 54, 88–109. 10.33594/000000207 31990489

[B31] Meng X. M. Tang P. M. Li J. Lan H. Y. (2015). TGF-β/Smad signaling in renal fibrosis. Front. Physiol. 6, 82. 10.3389/fphys.2015.00082 25852569 PMC4365692

[B32] Miralbell R. Bieri S. Mermillod B. Helg C. Sancho G. Pastoors B. (1996). Renal toxicity after allogeneic bone marrow transplantation: the combined effects of total-body irradiation and graft-versus-host disease. J. Clin. Oncol. 14, 579–585. 10.1200/JCO.1996.14.2.579 8636774

[B33] Odudu A. Nery F. Harteveld A. A. Evans R. G. Pendse D. Buchanan C. E. (2018). Arterial spin labelling MRI to measure renal perfusion: a systematic review and statement paper. Nephrol. Dial. Transpl. 33, ii15–ii21. 10.1093/ndt/gfy180 30137581 PMC6106644

[B34] Olson J. D. Tooze J. A. Bourland D. J. Cline J. M. Faria E. B. Cohen E. P. (2023). Measurement of renal cortical fibrosis by CT scan. Res. Diagn Interv. Imaging 5, 100024. 10.1016/j.redii.2023.100024 37155521 PMC10124964

[B35] Ostermann M. Zarbock A. Goldstein S. Kashani K. Macedo E. Murugan R. (2020). Recommendations on acute kidney injury biomarkers from the acute disease quality initiative consensus conference: a consensus statement. JAMA Netw. Open 3, e2019209. 10.1001/jamanetworkopen.2020.19209 33021646

[B36] Patel V. Noureddine L. (2012). MicroRNAs and fibrosis. Curr. Opin. Nephrol. Hypertens. 21, 410–416. 10.1097/MNH.0b013e328354e559 22622653 PMC3399722

[B37] Peters A. M. (2025). Renography: methods and pitfalls. Semin. Nucl. Med. 10.1053/j.semnuclmed.2025.11.001 41365767

[B38] Petzuch B. Bénardeau A. Hofmeister L. Meyer J. Hartmann E. Pavkovic M. (2022). Urinary miRNA profiles in chronic kidney injury-benefits of extracellular vesicle enrichment and miRNAs as potential biomarkers for renal fibrosis, glomerular injury, and endothelial dysfunction. Toxicol. Sci. 187, 35–50. 10.1093/toxsci/kfac028 35244176

[B39] Pruijm M. Milani B. Pivin E. Podhajska A. Vogt B. Stuber M. (2018). Reduced cortical oxygenation predicts a progressive decline of renal function in patients with chronic kidney disease. Kidney Int. 93, 932–940. 10.1016/j.kint.2017.10.020 29325997

[B40] Qin W. Chung A. C. Huang X. R. Meng X. M. Hui D. S. Yu C. M. (2011). TGF-β/Smad3 signaling promotes renal fibrosis by inhibiting miR-29. J. Am. Soc. Nephrol. 22, 1462–1474. 10.1681/ASN.2010121308 21784902 PMC3148701

[B41] Raavi V. Perumal V. Paul S. F. D. (2021). Potential application of γ-H2AX as a biodosimetry tool for radiation triage. Mutat. Res. Rev. Mutat. Res. 787, 108350. 10.1016/j.mrrev.2020.108350 34083048

[B42] Rabadi T. Kirchner L. Pausch A. M. Elsner C. Hötker A. M. (2025). Influence of age on parameters of multiparametric functional renal MRI: a prospective exploratory study. Eur. J. Radiol. 192, 112366. 10.1016/j.ejrad.2025.112366 40865256

[B43] Rodríguez-Ribera L. Corredor Z. Sandoval S. B. Coll E. Silva I. Diaz J. M. (2015). Radiosensitivity in patients suffering from chronic kidney disease. Int. J. Radiat. Biol. 91, 172–178. 10.3109/09553002.2015.959670 25219678

[B44] Rosiello R. A. Merrill W. W. (1990). Radiation-induced lung injury. Clin. Chest Med. 11, 65–71. 2182279

[B45] Sharma M. Halligan B. D. Wakim B. T. Savin V. J. Cohen E. P. Moulder J. E. (2008). The urine proteome as a biomarker of radiation injury: submitted to Proteomics- clinical applications special issue: renal and urinary proteomics (thongboonkerd). Proteomics Clin. Appl. 2, 1065–1086. 10.1002/prca.200780153 19746194 PMC2739391

[B46] Shatta A. E. Mostafa M. A. Attia M. A. Zaho T. A. Kazibwe R. Soliman E. Z. (2025). Impaired kidney function, subclinical myocardial injury, and their joint associations with cardiovascular mortality in the general population. J. Clin. Med. 14, 7123. 10.3390/jcm14197123 41096203 PMC12525244

[B47] Siew K. Nestler K. A. Nelson C. D'Ambrosio V. Zhong C. Li Z. (2024). Cosmic kidney disease: an integrated pan-omic, physiological and morphological study into spaceflight-induced renal dysfunction. Nat. Communications 15, 4923. 10.1038/s41467-024-49212-1 38862484 PMC11167060

[B48] Solana-Peña Á. Pujol-Canadell M. Macià M. Pérez E. M. Linares I. Stefanovic M. (2025). Evaluation of predictive biomarkers for late radiation toxicity in breast cancer patients. Sci. Rep. 16, 1366. 10.1038/s41598-025-31033-x 41331053 PMC12796360

[B49] Stabinska J. Piccolo J. Chhabra A. Liatsou I. Gabrielson K. Li Z. (2025). MRI detects tubulointerstitial changes in mouse models of radiation-induced nephropathy. Magn. Reson Med. 94, 251–261. 10.1002/mrm.30443 39846230 PMC12052301

[B50] Steinhelfer L. Lunger L. Cala L. Pfob C. H. Lapa C. Hartrampf P. E. (2024). Long-term nephrotoxicity of (177)Lu-PSMA radioligand therapy. J. Nucl. Med. 65, 79–84. 10.2967/jnumed.123.265986 37857504

[B51] Street J. M. Koritzinsky E. H. Glispie D. M. Yuen P. S. T. (2017). Urine exosome isolation and characterization. Methods Mol. Biol. 1641, 413–423. 10.1007/978-1-4939-7172-5_23 28748478 PMC9374111

[B52] Sugiyama K. Inoue T. Kozawa E. Ishikawa M. Shimada A. Kobayashi N. (2020). Reduced oxygenation but not fibrosis defined by functional magnetic resonance imaging predicts the long-term progression of chronic kidney disease. Nephrol. Dial. Transpl. 35, 964–970. 10.1093/ndt/gfy324 30418615

[B53] Taylor A. T. Brandon D. C. de Palma D. Blaufox M. D. Durand E. Erbas B. (2018). SNMMI procedure Standard/EANM practice guideline for diuretic renal scintigraphy in adults with suspected upper urinary tract obstruction 1.0. Semin. Nucl. Med. 48, 377–390. 10.1053/j.semnuclmed.2018.02.010 29852947 PMC6020824

[B54] Tepus M. Tonoli E. Verderio E. A. M. (2022). Molecular profiling of urinary extracellular vesicles in chronic kidney disease and renal fibrosis. Front. Pharmacol. 13, 1041327. 10.3389/fphar.2022.1041327 36712680 PMC9877239

[B55] Tournebize C. Schleef M. De Mul A. Pacaud S. Derain-Dubourg L. Juillard L. (2025). Multiparametric MRI: can we assess renal function differently? Clin. Kidney J. 18, sfae365. 10.1093/ckj/sfae365 40008350 PMC11852294

[B56] Tran L. K. Maturen K. E. Feng M. U. Wizauer E. J. Watcharotone K. Parker R. A. (2014). Renal remodeling after abdominal radiation therapy: parenchymal and functional changes. AJR Am. J. Roentgenol. 203, W192–W198. 10.2214/AJR.13.12149 25055293

[B57] Verginadis I. I. Citrin D. E. Ky B. Feigenberg S. J. Georgakilas A. G. Hill-Kayser C. E. (2025). Radiotherapy toxicities: mechanisms, management, and future directions. Lancet 405, 338–352. 10.1016/s0140-6736(24)02319-5 39827884 PMC12758832

[B58] Virzì G. M. Morisi N. Oliveira Paulo C. Clementi A. Ronco C. Zanella M. (2025). Neutrophil gelatinase-associated lipocalin: biological aspects and potential diagnostic use in acute kidney injury. J. Clin. Med. 14, 1570. 10.3390/jcm14051570 40095516 PMC11900132

[B59] Wang N. Zhou Y. Jiang L. Li D. Yang J. Zhang C. Y. (2012). Urinary microRNA-10a and microRNA-30d serve as novel, sensitive and specific biomarkers for kidney injury. PLoS One 7, e51140. 10.1371/journal.pone.0051140 23272089 PMC3521774

[B60] Wang W. Zaho T. Cao A. Badlani G. H. Rais-Bahrami S. Atala A. (2026). Regenerative strategies for post-prostatectomy incontinence: stem cells, exosomes, and the path to clinical resolution. Am. J. Clin. Exp. Urol. 14, 17–33. 10.62347/MBLA4964 41867505 PMC13003248

[B61] Wolf M. de Boer A. Sharma K. Boor P. Leiner T. Sunder-Plassmann G. (2018). Magnetic resonance imaging T1-and T2-mapping to assess renal structure and function: a systematic review and statement paper. Nephrol. Dial. Transpl. 33, ii41–ii50. 10.1093/ndt/gfy198 30137583 PMC6106643

[B62] Wu L. Boer K. Woud W. W. Udomkarnjananun S. Hesselink D. A. Baan C. C. (2021). Urinary extracellular vesicles are a novel tool to monitor allograft function in kidney transplantation: a systematic review. Int. J. Mol. Sci. 22, 10499. 10.3390/ijms221910499 34638835 PMC8508981

[B63] Yang T. K. Wang Y. J. Li H. J. Yu Y. F. Huang K. W. Cheng J. C. (2024). Efficacy and safety of hyperbaric oxygen therapy for radiation-induced hemorrhagic cystitis: a systematic review and meta-analysis. J. Clin. Med. 13, 4724. 10.3390/jcm13164724 39200867 PMC11355076

[B64] Zaho T. Soliman M. Z. Mostafa M. A. Shatta A. E. Attia M. A. Elbadawy M. S. (2025). Renal function, atrial cardiopathy, and their joint association with mortality in the general population. J. Clin. Med. 15, 122. 10.3390/jcm15010122 41517373 PMC12786734

[B65] Zhang Y. Jin D. Zhu H. Lin M. Peng X. (2025). Hepatocytes and hepatic stellate cells carry different levels of DNA damage due to their sensitivity to oxidative stress in chronic hepatitis B. J. Viral Hepat. 32, e70017. 10.1111/jvh.70017 39991880

